# Ishophloroglucin A, a Novel Phlorotannin for Standardizing the Anti-α-Glucosidase Activity of *Ishige okamurae*

**DOI:** 10.3390/md16110436

**Published:** 2018-11-08

**Authors:** BoMi Ryu, Yunfei Jiang, Hyun-Soo Kim, Jee-Min Hyun, Sang-Bin Lim, Yong Li, You-Jin Jeon

**Affiliations:** 1 Department of Marine Life Science, Jeju National University, Jeju 63243, Korea; ryu.bomi@gmail.com (B.R.); jiangyunfei0310@gmail.com (Y.J.); gustn783@naver.com (H.-S.K.); localman@jejunu.ac.kr (J.-M.H.); 2 Department of Food Bioengineering, Jeju National University, Jeju 63243, Korea; sblim@jejunu.ac.kr; 3 Department of Pharmaceutical Sciences, Changchun University of Chinese Medicine, 1035 Boshuo Road, Jing Yue Economic Development Zone, Chanchun 130117, China

**Keywords:** *Ishige okamurae*, Ishophloroglucin A, α-glucosidase inhibitory activity, validation

## Abstract

Nutraceutical use of algae requires understanding of the diversity and significance of their active compositions for intended activities. *Ishige okamurae* (*I. okamurae*) extract is well-known to possess α-glucosidase inhibitory activity; however, studies are needed to investigate its active composition in order to standardize its α-glucosidase inhibitory activity. In this study, we observed the intensity of the dominant compounds of each *I. okamurae* extract harvested between 2016 and 2017, and the different potency of each *I. okamurae* extract against α-glucosidase. By comparing the anti-α-glucosidase ability of the dominant compounds, a novel Ishophloroglucin A with highest α-glucosidase inhibitory activity was identified and suggested for standardization of anti-α-glucosidase activity in *I. okamurae* extract. Additionally, a validated analytical method for measurement of Ishophloroglucin A for future standardization of *I. okamurae* extract was established in this study. We suggest using Ishophloroglucin A to standardize anti-α-glucosidase potency of *I. okamurae* and propose the significance of standardization based on their composition for effective use of algae as marine-derived nutraceuticals.

## 1. Introduction

Development of novel nutraceuticals using marine alga or its composition has been the subject of intense research. Chemical composition of marine alga is shown to be altered by environmental changes including temperature, light, pH, and salinity [1,2]. It is, therefore, important to investigate the diversity of marine alga composition in the process of developing novel nutraceuticals. This is also important in order to standardize their potency based on their composition diversity and abundance.

*Ishige okamurae* (*I. okamurae*, a brown edible alga) distributes throughout the temperate coastal zone of Jeju in South Korea [3]. *I. okamurae* acts as an inhibitor of α-glucosidase, a key enzyme in the modulation of glucose absorption, and is considered a promising target for suppressing postprandial hyperglycemia [4,5,6]. The algae harbor a diverse chemical composition of different natures, among which vitamins and carotenoids are present within the fat-soluble fraction, and phlorotannins and phycobiliproteins are the most powerful water-soluble antioxidants present [7,8,9]. Among these compositions, phlorotannins, which have thus far been detected only in the form of marine alga polyphenols, are organic polymers of phloroglucinol (1,3,5-trihydroxybenzene).

Enabling commercial nutraceutical use of *I. okamurae* requires determination of its chemical composition and their biological activity. This is also important for standardization of the potency of *I. okamurae* as a nutraceutical under validated methods. We, therefore, monitored the chemical composition of each *I. okamurae* harvested from 2016 to 2017, and assessed the potency of *I. okamurae* extract against α-glucosidase. Furthermore, in this study, a predominant compound in *I. okamurae* extracts was newly identified to be a novel polyphenol-compound, Ishophloroglucin A (MW = 1986.26), with high α-glucosidase inhibitory activity. To standardize the activity of *I. okamurae* extract based on the quantitative difference of its predominant compound, Ishophloroglucin A, an HPLC analytical method was developed by validating the parameters of system suitability, linearity, precision, recovery, and reproducibility [10,11,12,13].

Accordingly, the main aims of this research were to address (1) the comparison of predominant compounds from each *I. okamurae* extract harvested between 2016 and 2017, (2) the identification of a novel compound, Ishophloroglucin A with high anti-α-glucosidase activity, and (3) development of a reliable validated analytical method for management and application of *I. okamurae.*

## 2. Results and Discussion

### 2.1. Composition of I. okamurae

Marine alga metabolism concerns the biochemical and transport processes by which algae absorb nutrients and convert them into materials needed for growth, reproduction and defense of the organism linked to particular environmental conditions or developmental stages [14,15].

The composition of marine alga produced by the defense metabolite has revealed important chemical prototypes as potential new therapeutic agents, stimulating the use of sophisticated physical techniques and new syntheses of compounds with biomedical application [16]. It is, therefore, required to assess marine alga composition prior to use in nutraceuticals [17,18]. Although *I. okamurae* is an edible brown alga which has inhibitory activity against α-glucosidase, no research has yet been conducted to standardize this activity based on the variety in its composition in order to define potential application [19,20,21]. In this study, *I. okamurae* was collected at four different times from Seongsan areas along the Jeju coastline from April 2016 to June 2017 (Table 1).

**Figure 1:**
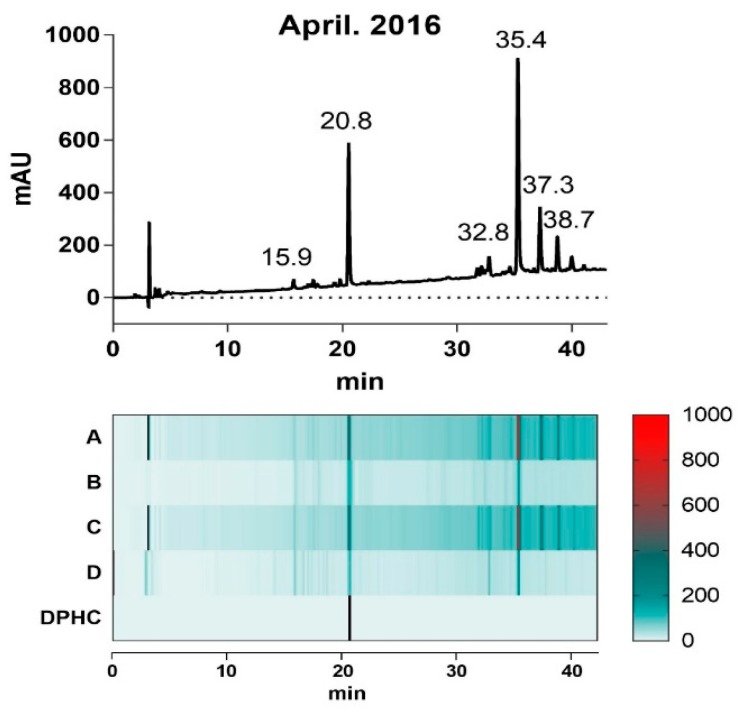
Chromatogram profile of the *I. Okamurae* harvested in April of 2016 (upper layer) acquired by Agilent HPLC system (230 nm), and heatmap matrix displaying the relative area levels of profile of the four extracts harvested as indicated in Table 1.

The ethanol extracts of each collected *I. okamurae* (A–D) appeared to have similar presence of HPLC chromatogram profiles of the predominant bimodal pattern. However, the intensity of the dominant peaks showed variable levels depending on the harvest time (Figure 1); the relative intensity of the peak at 20.8 min and 35.4 min, respectively, in extracts (A) and (C) were remarkably reduced in (B) and (D).

To investigate the correlation between the intensity of the two predominant peaks in each extract and their α-glucosidase inhibitory activity, we evaluated the IC_50_ values of each extract in inhibition of α-glucosidase activity (Table 1). Interestingly, the difference in α-glucosidase inhibitory activity of each extract was proportional to its area level of each peaks at 20.8 min and 35.4 min, suggesting that the different contents of the components in each extract can presumably be responsible for the divergent potency of the extracts in inhibiting α-glucosidase activity.

The mass spectrum of each extract (A–D) were aligned and their isolates were systemically compared based on previous study of identification of *I. okamurae* extract composition (Supplementary Figure S1). DPHC (diphlorethohydroxycarmalol, 512.06 g/mol) that was detected at 20.8 min, is a phlorotannin derived from *I. okamurae* with anti-diabetes activity related to its α-glucosidase inhibitory activity [19], however, a mass of 1986.26 g/mol that was detected at 35.4 min was yet to be identified. In this study, we hypothesized that the content of the dominant compositions of *I. okamurae* can explain the α-glucosidase inhibitory activity of the extract.

### 2.2. Identification of Ishophloroglucin A

The mass of 1986.26 g/mol that was present in all the extracts of *I. okamurae* was further characterized. Two-phase solvent system of preparative centrifugal partition chromatography (CPC) according to their affinity to each of the phases in a given biphasic solvent system was subjected in order to separate the composition of ethanol extract [22,23,24]. The partition coefficient *K*, critical for a successful CPC separation, was expressed by the amount of solute in the stationary phase divided by that of the mobile phase [25]. The *K* value of the compound was calculated in the solvent system of n-hexane:ethyl acetate:methanol:water (1:9:4.5:6.5, *v*/*v*), and showed as 0.50, which was within the acceptable range of 0.50 to 1.0. The compound was further isolated in isocratic mode of semi-prep column and its mass was analyzed with the extracted ion chromatogram (EIC) by UHPLC-Q-ToF. LC/MS analysis of this compound showed a mass *m*/*z* of 992.1315, indicating a molecular formula of C_96_H_66_O_48_ (1986.26 of calculated molecular weight, ∆ 0.6, [M − 2H]^2−^), whereby spacing of the ^13^C isotope peaks of *m*/*z* 0.5 indicates a doubly-charged ion.

Furthermore, the NMR data for HMQC and HMBC of the compound is shown in Supplementary Figures S2 and S3 and the chemical shift for identifying the compound is suggested in Table 2.

**Figure 2:**
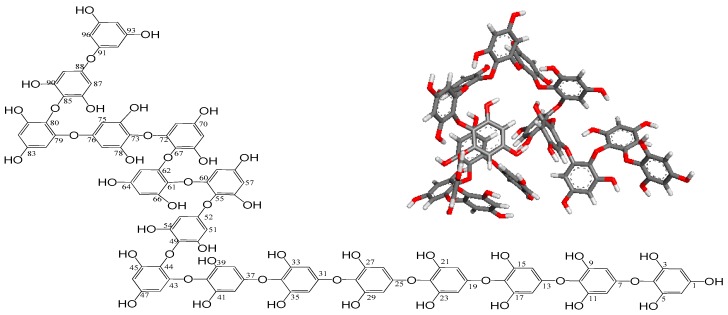
Chemical structure and 3D structure (inner panel) of the Ishophloroglucin A. The 3D structure of Ishophloroglucin A was obtained from Gaussian 09 program using the semi empirical PM6 method.

These NMR data suggest that the compound is a polyphenol-compound possessing repeated phenolic hydroxyl groups. On the basis of its molecular formula from LC/MS and ^1^H and ^13^C NMR data, its unsaturated degree is expected to be 64 from 16 aromatic rings (four for each). Given the appearance of carbon signals in only three configurations at around 150 ppm, 120 ppm, and 90 ppm from the ^13^C NMR spectrum, it is evident that the chemical structure of the compound should be polyphenol including 16 phenol homopolymers with a maximum characteristic absorption at 230 nm (benzene ring sp^2^ π bond). The linkages among these 16 phenols is expected to be single-bonded without any rings, as well as double-bonded. No signals were detected from 100 ppm to 150 ppm with the exception of 120 ppm carbon atoms in the ^13^C spectrum, indicating that the linkage forms among 16 phenols meet to three configurations consisting of: extraneous oxygen atoms binding to two aromatic carbon atoms directly (providing the chemical shifts at around 120 ppm), linkages containing endogenous oxygen atoms from two phenolic hydroxyl groups of phenol units by dehydration, and linkages with oxygen bridge atoms from endogenous phenol –OH directly bonded to aromatic carbon atom clearly. According to the data of ^1^H, ^13^C, HMQC, and HMBC NMR spectra, the signals from δ_H_ 8.92–9.07 ppm in ^1^H NMR spectrum were elucidated as proton atoms from free phenol hydroxyl groups and the other protons of 5.59–6.15 ppm meet to the free aromatic hydrogen atoms. In 13C NMR data, the chemical shift at 94.1–94.9 ppm presented the aromatic carbons. After the comprehensive analyses of all these spectra data and SciFinder database, the structure of compound was determined as a novel polyphenol named as Ishophloroglucin A with unique complex linkages (Figure 2).

### 2.3. α-Glucosidase Inhibitory Activity of Ishophloroglucin A

To understand the association between abundance of Ishophloroglucin A and DPHC of *I. okamurae* and the α-glucosidase inhibitory effect of each extract (A–D), each compound was investigated for its inhibition of α-glucosidase activity. As shown in Table 3, Ishophloroglucin A exhibited IC_50_ value of 54.97 µM in α-glucosidase inhibition, three-times lower than that of DPHC (IC_50_ 175.78 µM), while the acarbose (a commercial digestive enzyme inhibitor) showed an IC_50_ of 1.05 mM, indicating that Ishophloroglucin A could be a key component for the α-glucosidase inhibitory activity of the *I. okamurae* extract. Therefore, Ishophloroglucin A with high α-glucosidase inhibitory activity can explain the potency of *I. okamurae* extract against α-glucosidase, and can be an index substance to represent and standalize the capacity of the extract for its α-glucosidase activity.

### 2.4. Method Validation

Here, Ishophloroglucin A with strong α-glucosidase inhibitory activity was used to standalize the α-glucosidase activity of *I. okamurae* using the validated method below.

The validation procedure included all the necessary steps to ensure correct identification of Ishophloroglucin A and appropriate quantification, and involved the examination of the following parameters: system suitability, linearity, precision, recovery and reproducibility. The system suitability test was used to verify if the resolution and reproducibility of the system are adequate for the analysis [26]. The RSD (%) of retention time (*RT*) and peak area (*Pa*) for Ishophloroglucin A standards were 0.75% and 3.09%, respectively (Table 4). Additionally, the capacity factor (*K’*) value, the efficiency of the column as expressed with plate number (*N*), tailing factor (*Tf*), and the baseline separation expressed as resolution (*Rs*) all met the requirements stated in United States Pharmacopeia (USP) (*K’* > 2.0, *N* > 2000, 0.5 ≤ *Tf* ≤ 2 and *Rs* > 2) for reliability of the analytical condition of Ishophloroglucin A in *I. okamurae* [26,27].

The regression statistics were calculated from the calibration curves constructed for all the studied Ishophloroglucin A and showed satisfactory linearity with the correlation coefficient (*R*^2^) of greater than 0.999 (Table 5). Based on the obtained regression curve, the concentrations of Ishophloroglucin A in each *I. okamurae* extract in Table 1 from April 2016 to June 2017 were measured as 67.26, 25.80, 61.08, and 19.30 µg/mL, which is proportional to the α-glucosidase inhibitory activity levels in their extract.

The sensitivity of the method was expressed as limit of detection (LOD) and limit of quantification (LOQ), using the RSD of the regression line (*σ*) and slope (*S*) of the calibration curve for Ishophloroglucin A standard with LOD of 3.3 *σ*/*S* and LOQ of 10 *σ*/*S* [27]. The method proved to be sensitive for Ishophloroglucin A detection and quantification with the limit of as low as 3.8 and 11.5 µg/mL respectively (Table 5).

Precision was evaluated by performing intra- and inter-day repeatability studies through analyzing the closeness of agreement between the result of a measurement and a true value. *I. okamurae* extract (0.5 mg/mL, 1 mg/mL and 5 mg/mL) was injected three times and RSD values of Ishophloroglucin A were calculated. As Table 6 shows, the RSDs of RT and Pa for both intra- and inter-day variability of Ishophloroglucin A were within the acceptable range of 2%, indicating stability of the utilized method. The % recovery of Ishophloroglucin A (mean between 94.25%–99.52%) was found to be in between the predefined acceptance criterion of 80% and 120.0% [27]. Additionally, the obtained values for the RSD (*n* = 3) of the *RT* and *Pa* of Ishophloroglucin A in two different HPLC systems were in accordance with this criterion (<2%).

## 3. Materials and Methods

### 3.1. Reagents and Chemicals

HPLC grade acetonitrile and methanol were obtained from Honeywell Burdick and Jackson (Muskegon, MI, USA). Analytical-grade formic acid was purchased from Fluka Chemica (Buchs, Switzerland) and water was filtered by the Milli Q system (Millipore, Milford, MA, USA). All other chemicals used were of analytical grade.

### 3.2. Collection of I. okamurae

*I. okamurae* samples were collected four times between June 2016 to June 2017, from Seongsan along the Jeju coastline in South Korea and were de-salted/dried and stored at −20 °C until needed (details in Table 1). The marine alga extracts were prepared by a modified original procedure described previously in our earlier paper [19].

### 3.3. Spectrometric Analyses

The chromatographic analyses were conducted on an Agilent 1260 Infinity II gradient LC system VL (Agilent Technologies, Palo Alta, CA, USA) equipped with an Agilent poroshell 120 EC-C18 column (4.6 mm × 150 mm, 4 μm) and a UV detector (230 nm). The mobile phase consisted of (A) 0.1% formic acid in water and (B) ACN containing 0.1% formic acid. The HPLC eluting conditions were as follows: 5–40% B for 40 min to 100% B for 10 min, followed by 10 min re-equilibration time of the column. The flow rate was maintained at 0.3 mL/min and the injected volume was 10 μL. Mass spectrometric analyses were obtained using a SYNAPT G2-Si HDMS (Waters) equipped with an electrospray ionization (ESI) source (Korea basic science institute (KBSI), Seoul, South Korea). ESI mass spectra were acquired over the *m*/*z* 100–2000 range. The capillary voltage and the sampling cone voltage were set to −2 kV and −30 V at source temperature of 300 °C, respectively. Spectra were acquired in continuum and negative mode. Argon was employed as the collision gas. Analysis was performed by the data dependent method as follow: (i) full-scan MS in the m/z range 100–2000; (ii) zoom scan of the two most intense ions. Blank runs confirmed that there was no carry-over of compounds between runs. The SYNAPT G2-Si HDMS system was calibrated using sodium formate clusters and operated in resolution mode. Repeated injections of the same extract confirmed that between runs in retention time were within ±1 min and reconstructed masses were within ±0.1 Da.

### 3.4. Isolation of Ishophloroglucin A

The ethanol extract (Lot No. SW8D10SA, provided by Shinwoo Co., LTD, Uiwang, South Korea) was fractionated using centrifugal partition chromatography (CPC 240, Tokyo, Japan). The rotor had a total volume of 1 L. The two-phase solvent system was composed of n-hexane:ethyl acetate:methanol:water (1:9:4.5:6.5, *v*/*v*). Elution mode was descending with the organic phase acting as the stationary phase and the aqueous phase as the mobile phase. The extract (500 mg) was dissolved in 6 mL of water:methanol (1:1, *v*/*v*) of CPC solvent phase and delivered by an isocratic pump (Kromaton). The effluent from the outlet of the column was continuously monitored by a UV detector at 230 nm and collected into test tubes with a fraction collector set at 3 min for each tube. All fractions of the same compound were combined for further purification by semipreparative HPLC column (YMC-Pack ODS-A, 10 mm × 250 mm, 5 μm), eluting with an isocratic system of 32% acetonitrile containing 0.1% formic acid (Sigma-Aldrich, St. Louis, MO, USA) at a flow rate of 2 mL/min.

NMR spectra were recorded in DMSO-*d*_6_ on a Bruker Biospin Advance II 900 NMR spectrometer at Korea Basic Science Institute (KBSI) in Ochang, South Korea. Resonance assignment was achieved by two-dimensional homo and heteronuclear correlation NMR spectra (HMQC and HMBC). Chemical shifts for the isolated compound are shown as d-values and peak multiplicities are quoted in Hz.

Ishophloroglucin A (99%): light yellowish solid; ^1^H NMR (DMSO-*d*_6_, 900 MHz) and ^13^C NMR (DMSO-*d*_6_, 226 MHz) records were shown in HMQC and HMBC data (Supplementary Figures S2 and S3). MS spectrum in negative mode for [M − 2H]^2−^ at *m*/*z* 992.1284 (*z* = 2, calcd for C_96_H_66_O_48_, *m* = 1986.2568); 4-(3,5-dihydroxyl phenoxy)-2-(2,6-dihydroxyl phenoxy)-4-(3,5-dihydroxyl phenoxy)-2-(4,6-dihydroxyl phenoxy)3-(4,6-dihydroxyl phenoxy)-(2,6-dihydroxyl phenoxy)-4-(3,5-dihydroxyl phenoxy)6-1,3,5-phloroglucin.

### 3.5. Measurement of α-Glucosidase Inhibitory Activity

The α-glucosidase inhibitory assay was conducted using chromogenic method described by Watanabe et al. [28] using a readily available yeast enzyme. The inhibition was measured spectrophotometrically at pH 7.0 and 37 °C using 5 mM p-nitrophenyl α-d-glucopyranoside (PNP-G) as a substrate and 32 mU/mL of enzyme (yeast α-glucosidase, Sigma) in 100 mM phosphate buffer (enzyme stock). The absorbance of 4-nitrophenol released by the hydrolysis of PNP-G was measured at 405 nm as time zero record. After incubation for 5 min, equal volume of substrate solution was added and incubated for another 5 min at room temperature. The increase in the absorbance from time zero was measured. Percent inhibitory activity was expressed as 100 minus relative absorbance differences (%) of test compounds to absorbance change of the control where the test solution was replaced by carrier solvent.

### 3.6. Method Validation

The applicability of the developed method was tested following the accepted criteria for analytical method validation: system suitability and sensitivity, linearity, precision, accuracy, and recovery [12,13]. System suitability was evaluated by taking data from triplicate of the Ishophloroglucin A standard at a concentration of 1 mg/mL and resolution was acquired by analysis of Ishophloroglucin A in the ethanol extraction of *I. okamurae* at a concentration of 5 mg/mL. Parameters used for monitoring system suitability included relative standard deviation (RSD, %) of retention time (*RT*) and peak area (*Pa*), tailing factor (*Tf*), plate number (*N*), resolution (*Rs*), and capacity factor (*K′*), all calculated using the United States Pharmacopeia (USP) equations [26,27]. Sensitivity was assessed by measuring limit of detection (LOD) and limit of quantification (LOQ). The linearity of the method was assessed by conducting linear regression analysis. Eight concentrations of Ishophloroglucin A, over an expected concentration range of Ishophloroglucin A in *I. okamurae* extract, were used to construct the calibration curves. To assess the precision of the method, repeatability within intra- and inter-day runs was checked by calculating the RSD (%) for the *RT* and *Pa* of three replicate injections. Recovery was determined by spiking known quantities of Ishophloroglucin A into *I. okamurae* extract, at three different concentration levels within the analytical concentration range of the curve. Method reproducibility was evaluated by performing the analytical method on two HPLC systems within the lab: Instrument 1 was an Agilent 1260 Infinity II gradient LC system VL with UV detector (230 nm) operated with ChemStation, and Instrument 2 was a Waters HPLC system with two mono Waters 515 HPLC pumps, a Waters 2998 photodiode array detector (230 nm), a Waters 2707 autosampler, and the Waters pump control module II, and was operated with Empower (Waters Corporation, Milford, MA, USA). Chromatography was performed on an Agilent poroshell 120 C18 (4 μm, 4.6 mm × 150 mm).

### 3.7. Statistical Analysis

Data were displayed as mean ± SD from three independent experiments. Statistical analyses were calculated by Microsoft Excel or GraphPad Prism (version 7.03, GraphPad Software, San Diego, CA, USA). Data were analyzed statistically using two-way ANOVA and significant differences between treatment means were determined using Dunnett’s multiple range tests at the *p* < 0.05 levels.

## 4. Conclusions

In this study, we identified a novel polyphenol-compound Ishophloroglucin A from *I. okamurae* with highest α-glucosidase inhibitory activity, for use in standardization of the activity of *I. okamurae* against α-glucosidase for potential nutraceutical application.

Furthermore, for efficient and reliable management of the potency of *I. okamurae* extract with α-glucosidase inhibitory activity, we developed and validated an analytical method of Ishophloroglucin A from *I. okamurae* extract that may help to standardize the potency of *I. okamurae* extract.

## Figures and Tables

**Table 1:** Harvest details and α-glucosidase inhibitory activity of *I. okamurae.*

Species	Harvest Location	Extract	Harvest Time	IC_50_ α-Glucosidase Inhibition (mg/mL)
*Ishige Okamurae*	Seongsan, JeJu	A	April 2016	0.22 ± 0.05
		B	June 2016	0.48 ± 0.05
		C	March 2017	0.28 ± 0.06
		D	June 2017	0.52 ± 0.03

**Table 2:** The chemical shift of Ishophloroglucin A * (*m*/*z* = 992.1284, *z* = 2).

No	δ_H_ (Mult, *J*)	δ_C_ (mult)	No	δ_H_ (Mult, *J*)	δ_C_ (Mult)	No	δ_H_ (Mult, *J*)	δ_C_ (Mult)	No	δ_H_ (Mult, *J*)	δ_C_ (Mult)
1		156.2 (s)	26	5.85 (^1^H, s)	94.7 (d)	51	5.85 (^1^H, s)	94.7 (d)	76		154.0 (s)
2	5.95 (^1^H, d, *J* = 1.6 Hz)	94.2 (d)	27		151.1 (s)	52		154.0 (s)	77	5.85 (^1^H, s)	94.7 (d)
3		151.1 (s)	28		122.0 (s)	53	5.85 (^1^H, s)	94.7 (d)	78		151.1 (s)
4		122.0 (s)	29		151.1 (s)	54		151.1 (s)	79		152.7 (s)
5		151.1 (s)	30	5.85 (^1^H, s)	94.7 (d)	55		123.4 (s)	80		123.5 (s)
6	5.95 (^1^H, d, *J* = 1.6 Hz)	94.2 (d)	31		154.1 (s)	56		150.8 (s)	81		150.8 (s)
7		154.0 (s)	32	5.85 (^1^H, s)	94.7 (d)	57	5.60 (^1^H, d, *J* = 1.6 Hz)	94.1 (d)	82	5.70 (^1^H, d, *J* = 1.8 Hz)	94.9 (d)
8	5.85 (^1^H, s)	94.7 (d)	33		151.1 (s)	58		153.0 (s)	83		152.9 (s)
9		151.1 (s)	34		122.0 (s)	59	5.75 (^1^H, dd, J = 1.8, 1.6 Hz)	94.7 (d)	84	5.75 (^1^H, dd, *J* = 1.8, 1.6 Hz)	94.7 (d)
10		122.0 (s)	35		151.1 (s)	60		152.7 (s)	85		122.0 (s)
11		151.1 (s)	36	5.85 (^1^H, s)	94.7 (d)	61		123.4 (s)	86		151.1 (s)
12	5.85 (^1^H, s)	94.7 (d)	37		154.1 (s)	62		152.7 (s)	87	5.85 (1H,s)	94.7 (d)
13		154.1 (s)	38	5.85 (^1^H, s)	94.7 (d)	63	5.75 (^1^H, dd, *J* = 1.8, 1.6 Hz)	94.7 (d)	88		154.5 (s)
14	5.85 (^1^H, s)	94.7 (d)	39		151.1 (s)	64		153.0 (s)	89	5.85 (1H,s)	94.7 (d)
15		151.1 (s)	40		122.0 (s)	65	5.60 (^1^H, d, *J* = 1.6 Hz)	94.1 (d)	90		151.1 (s)
16		122.0 (s)	41		151.1 (s)	66		150.8 (s)	91		161.0 (s)
17		151.1 (s)	42	5.85 (^1^H, s)	122.0 (s)	67		123.4 (s)	92	6.15 (^2^H, d, *J* = 1.6 Hz)	94.9 (d)
18	5.85 (^1^H, s)	94.7 (d)	43		152.7 (s)	68		150.8 (s)	93		158.6 (s)
19		154.1 (s)	44		123.5 (s)	69	5.60 (^1^H,d, *J* = 1.6 Hz)	94.1 (d)	94	5.95 (^1^H, d, *J* = 1.6 Hz)	94.1 (d)
20	5.85 (^1^H, s)	94.7 (d)	45		150.8 (s)	70		153.0 (s)	95		158.6 (s)
21		151.1 (s)	46	5.70 (^1^H, d, *J* = 1.8 Hz)	94.9 (d)	71	5.75 (^1^H, dd, *J* = 1.8, 1.6 Hz)	94.7 (d)	96	6.15 (^2^H, d, *J* = 1.6 Hz)	94.9 (d)
22		122.0 (s)	47		152.9 (s)	72		152.7 (s)	–OH *	8.92–9.07 (33 H, m)	
23		151.1 (s)	48	5.75 (^1^H, dd, *J* = 1.8, 1.6 Hz)	94.7 (d)	73		122.0 (s)			
24	5.85 (^1^H, s)	94.7 (d)	49		122.0 (s)	74		151.1 (s)			
25		154.1 (s)	50		151.1 (s)	75	5.85 (^1^H, s)	94.7 (d)			

* Recorded in DMSO-*d*_6_ at 900 MHz for ^1^H NMR and 226 MHz for ^13^C NMR; –OH *: all free phenol hydroxyl groups.

**Table 3:** α-glucosidase inhibitory activity of Ishophloroglucin A, DPHC and acarbose.

Compound	IC_50_ α-Glucosidase Inhibition
Ishophloroglucin A	54.97 ± 0.06 µM
DPHC	175.78 ± 0.04 µM
Acarbose	1050.23 ± 0.09 mM

**Table 4:** System suitability parameters for validation of the developed method. The values are the average RSD of Ishophloroglucin A from three consecutive injections of *I. okamurae* (5 mg/mL).

*RT* (RSD)	*Pa* (RSD)	*K’*	*N*	*Tf*	*Rs*
0.75	0.13	5.19 ± 0.21	21,217.4	0.52	3.54 ± 0.03

*RT* = retention time; *Pa* = peak area; *K′* = capacity factor; *Tf* = tailing factor; *N* = plate number; *Rs* = resolution.

**Table 5:** Regression (sensitivity, linearity) statistics, limit of detection (LOD), and limit of quantification (LOQ) for the calibration curves of Ishophloroglucin A.

Concentration range (µg/mL)	Slope	Intercept	*R* ^2^	LOD (µg/mL)	LOQ (µg/mL)
10–1000	117.22	479.92	0.9999	3.80	11.50

*R*^2^ = the correlation coefficient.

**Table 6:** Precision, recovery and reproducibility results for Ishophloroglucin A in *I. okamurae* extracts. Relative standard derivation (RSD) for the retention time (RT) and peak are (Pa) of Ishophloroglucin A from three replicate injections of *I. okamurae*.

Concentration (mg/mL)	Precision				Recovery (Mean, %)	Reproducibility			
	Intra-Day		Inter-Day			Instrument 1		Instrument 2	
	RT	Pa	RT	Pa		RT	Pa	RT	Pa
5	0.75	0.13	0.05	0.62	99.52 ± 2.93	0.69	0.13	0.95	0.90
1	0.26	0.21	0.76	0.53	94.25 ± 3.64	0.45	0.11	0.96	0.55
0.5	0.03	0.04	0.13	0.87	95.76 ± 3.58	0.22	0.05	0.83	0.68

## Supplementary Materials

The following are available online at http://www.mdpi.com/1660-3397/16/11/436/s1, Figure S1. MS spectra of DPHC (A, 512.06 g/mol) and Ishophloroglucin A (B, 1986.26 g/mol) in negative mode, Figure S2. HMQC spectrum of Ishophloroglucin A in DMSO-*d*_6_, Figure S3. HMBC spectrum of Ishophloroglucin A in DMSO-*d*_6_.
