# Supplementary material for: Ishophloroglucin A, a Novel Phlorotannin for Standardizing the Anti-α-Glucosidase Activity of Ishige okamurae
Source: Mar Drugs. 2018 Nov 8;16(11):436. doi: 10.3390/md16110436 (PMC6266998; doi:10.3390/md16110436)
Supplement: Supplementary file 1 [file marinedrugs-16-00436-s001.pdf]

Supplementary Materials

Table of Contents

Figure S1: MS spectra of DPHC (A, 512.06 g/mol) and Ishophloroglucin A (B, 1986.26 g/mol) in negative mode. 2
Figure S2. HMQC spectrum of Ishophloroglucin A in DMSO-d_6. 3
Figure S3. HMBC spectrum of Ishophloroglucin A in DMSO-d_6. 4

(A)

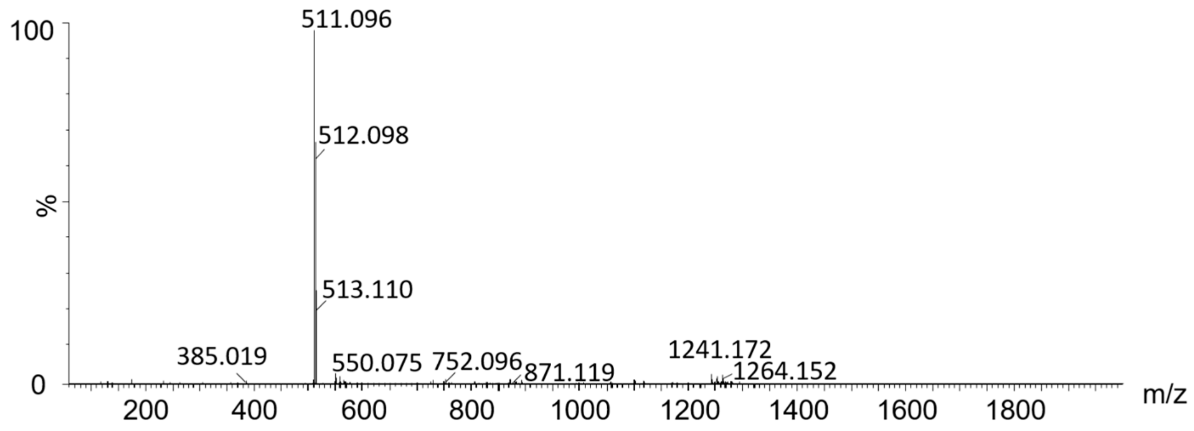

(B)

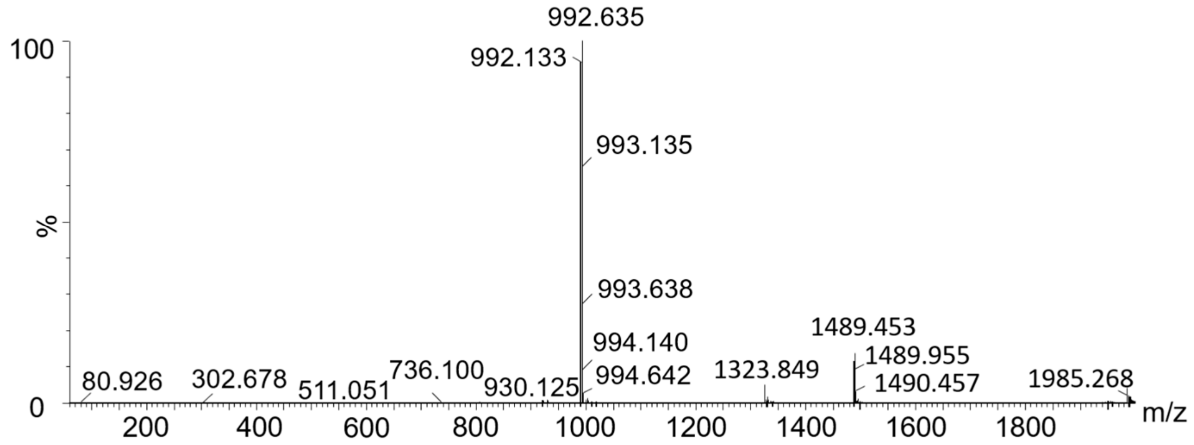

Figure S1. MS spectra of DPHC (A, 512.06 g/mol) and Ishophloroglucin A (B, 1986.26 g/mol) in negative mode.

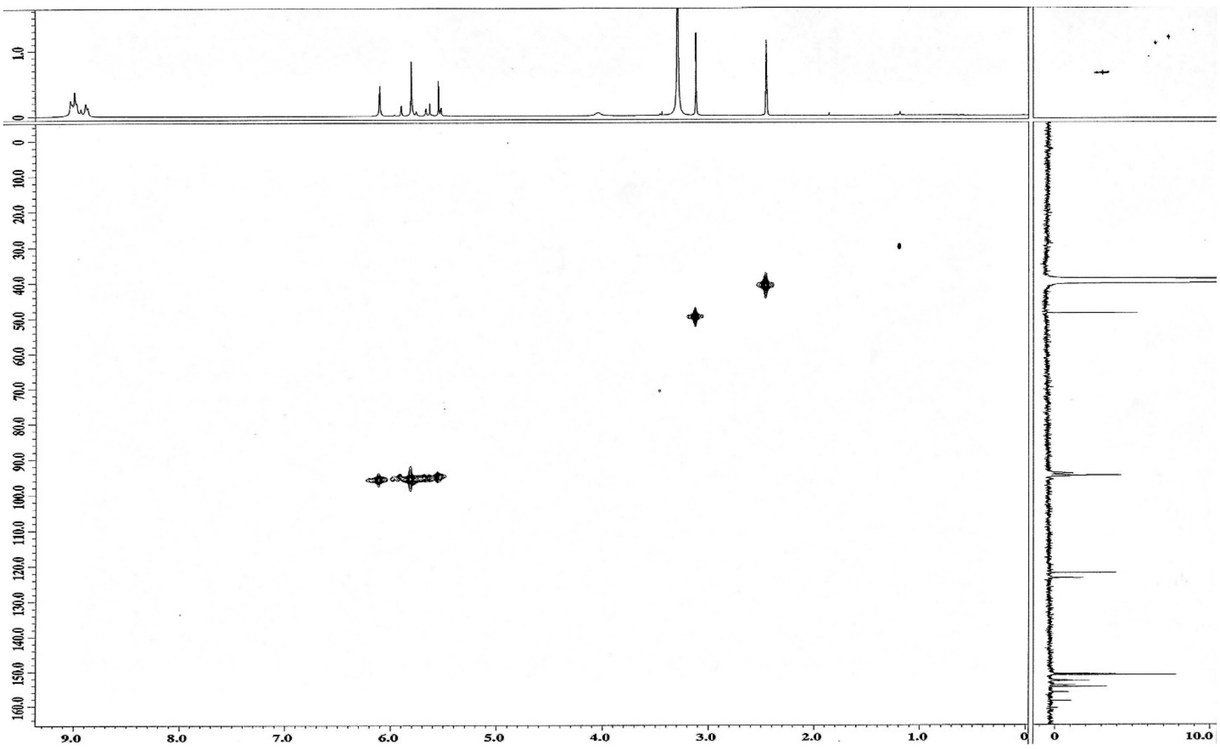

Figure S2. HMQC spectrum of Ishphloroglucin A in DMSO-*d*₆.

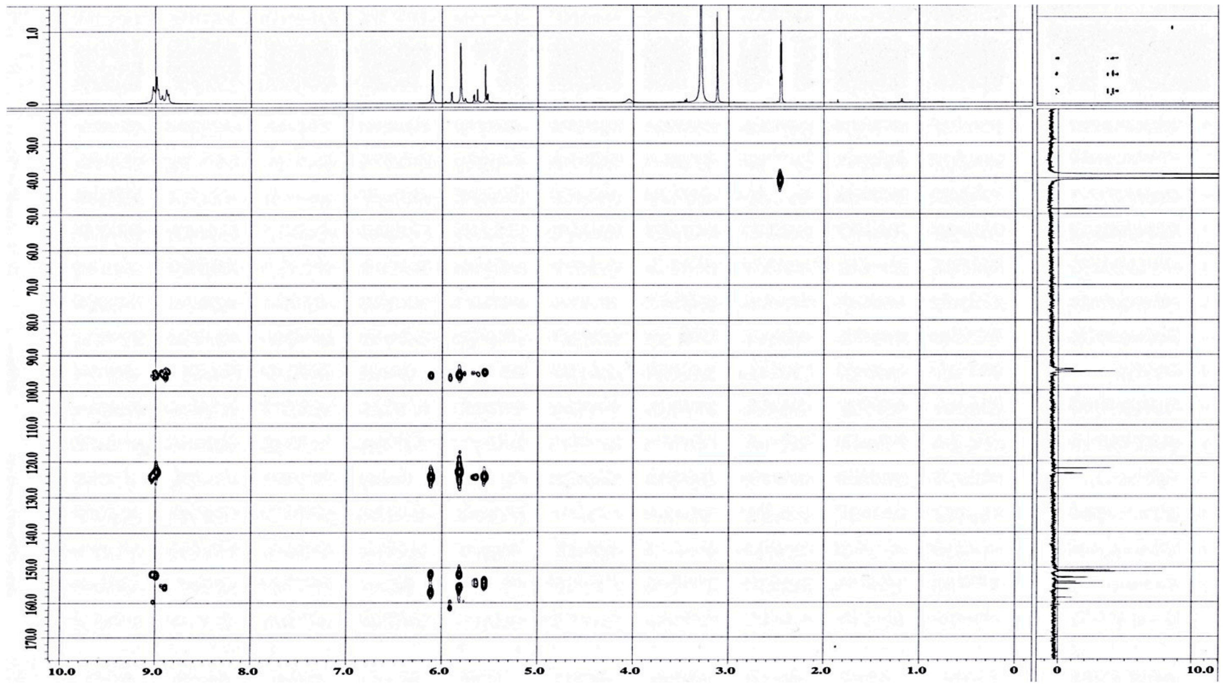

Figure S3. HMBC spectrum of Ishphloroglucin A in DMSO-d_6.
